# Towards the Development of Synthetic Routes Using Theoretical Calculations: An Application of *In Silico* Screening to 2,6-Dimethylchroman-4-one

**DOI:** 10.3390/molecules15118289

**Published:** 2010-11-15

**Authors:** Kenji Hori, Hirotaka Sadatomi, Atsuo Miyamoto, Takaaki Kuroda, Michinori Sumimoto, Hidetoshi Yamamoto

**Affiliations:** 1 Department of Material Science, Graduate School of Science and Technology, Yamaguchi University, Tokiwadai, Ube 755-8611, Japan; 2 Transition State Technology, Tokiwadai, Ube 755-8611, Japan; Email: sadatomi@tstcl.jp (H.S.); 3 NARD Institute, Ltd., Nishinagasu, Amagasaki 660-8050, Japan; Email: miyamoto@nard.co.jp (A.M.)

**Keywords:** *In silico* screening, synthetic route development, theoretical calculations, Mitsunobu reaction, Michael reaction

## Abstract

This study describes an attempt to develop a synthetic route using theoretical calculations, *i.e., in silico* synthesis route development. The KOSP program created four potential synthetic routes for generating 2,6-dimethylchroman-4-one. *In silico* screening of these four synthetic routes was then performed. *In silico* screening involves theoretical analysis of synthetic routes prior to actual experimental work. A synthetic route using the Mitsunobu reaction had already been reported by Hoddgets *et al*. Theoretical investigations were also conducted on two S_N_Ar reactions as well as a Michael reaction before they were examined experimentally. *In silico* screening using DFT calculations indicated that only the Michael reaction was likely to produce the target. Experimental work confirmed that the target was obtained in a yield of 76.4% using the Michael reaction. The other two routes, except for the Mitsunobu reaction, failed to generate the target. Our results demonstrate that theoretical calculations can be used to narrow down the number of experiments that need to be conducted when developing novel synthetic routes.

## 1. Introduction

Synthetic route design systems (SRDSs), such as LHAS [1], EROS [2] and AIPHOS [3], have been developed that make possible the creation of new synthetic routes for target compounds. The KOSP (Knowledgebase-Oriented Synthesis Planning) program [4,5], one of the AIPHOS family of programs, is now commercially available and has been used to create synthetic routes for a large number of targets. It is, therefore, possible to obtain synthetic routes without having detailed prior knowledge of the organic synthesis. However, there is still a significant degree of uncertainty using the current approaches, including the KOSP program. 

Theoretical calculations have been a very powerful tool to investigate mechanisms of chemical reactions. Transition state (TS) searches are the key to understanding reaction mechanisms in detail [6,7,8]. Using this approach we have been able to determine transition states (TSs) for existing chemical reactions. It is, therefore, likely that we can find TSs for synthetic reactions that have never been examined, *i.e.*, those reactions created by the SRDS. If there is a TS for a synthetic route, then it is feasible to use this information to design a method for synthesizing the target. However, if exhaustive efforts do not locate the TS then it is unlikely that the proposed reaction will produce the desired target. These computational analyses can be performed before starting any experimental work to synthesize the target compounds.

It is generally difficult to analyze reaction mechanisms from a theoretical perspective for synthetic routes that have not been examined experimentally, mainly due to a lack of reference data. Moreover, all possible reactions have to be analyzed, even though some of them are unwanted side reactions. In particular, it is necessary to check whether side reactions are likely to proceed by comparison with the main reaction. We have been investigating these computational procedures to analyze synthetic routes, which are referred to as “*in silico* screenings” [9,10,11].

**Scheme 1:**
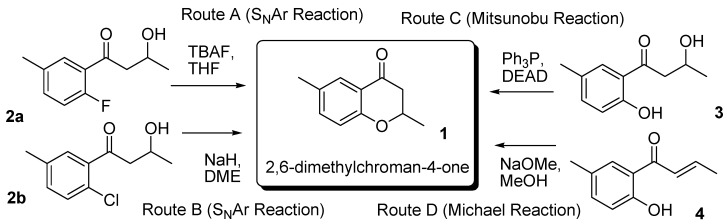
Synthetic Routes for 2,6-Dimethylchroman-4-one from the TOSP/KOSP program.

The present paper describes an *in silico* screening for synthetic routes with 2,6-dimethyl chroman-4-one (**1**) as the target compound. Comey originally extracted (*S*)-**1** from the roots of edelweiss (*Leontopodium alpinum*) [12]. The KOSP program generated more than twenty possible synthetic routes, which included using Pd-BINAP, the Mannich reaction of 1-(2-hydroxy-5-methylphenyl)ethanone with acetaldehyde, and other potential reactions [13]. Because it is difficult to perform *in silico* screening for such a large number of potential routes, we chose four such routes, as described in Scheme 1. Routes A and B use the S_N_Ar mechanism for the reactants with a hydroxyl group in the side chain and a halogen substituent on the aromatic ring.

Route C adopts the Mitsunobu reaction of a reactant **3** with aliphatic and aromatic hydroxyl groups [14]. The last synthetic route (Route D) uses the Michael reaction of **4** with a double bond in the side chain. The detailed mechanisms of these reactions will be discussed later. Although Route C had already been attempted experimentally, to the best of our knowledge the other routes had not been previously examined either theoretically or experimentally.

## 2. Results and Discussion

### 2.1. Routes A and B

**Scheme 2:**
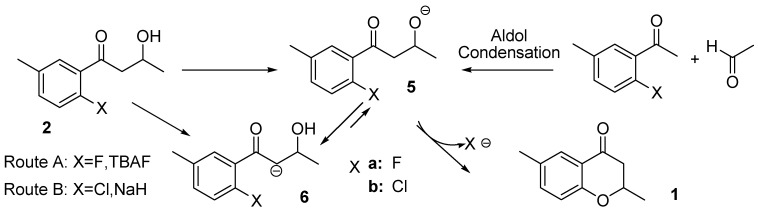
Plausible reaction pathways related to producing the target under a S_N_Ar mechanism.

In Route A, a fluorine anion from TBAF (tetrabutylammonium fluoride) acts as a base to extract a proton from the hydroxyl group of **2a** to form the alkoxide **5a**. Route B uses NaH to extract a proton from the alkoxyl group of **5b**. The target is then produced from the alkoxide under a S_N_Ar mechanism. Therefore, the reaction mechanism of Route A is almost the same as that of Route B shown in Scheme 2. The KOSP program suggested not only these reactions, but also the experimental conditions from papers describing similar reactions [15,16,17,18].

**Figure 1:**
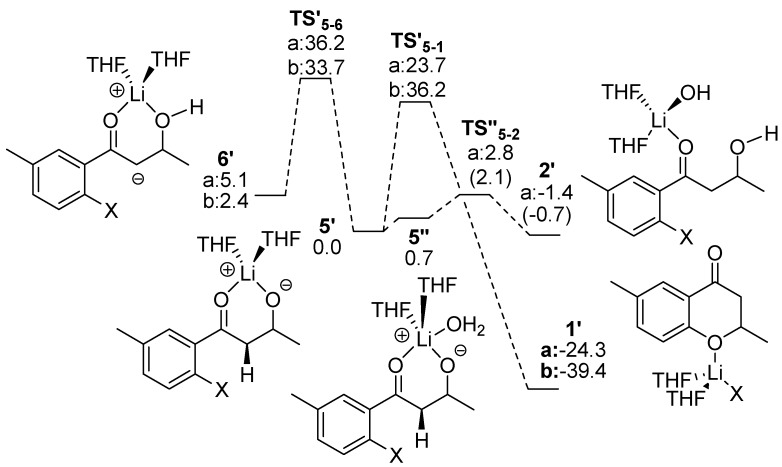
Energy correlation diagram (kcal mol^-1^) and TS structures (lengths in Å) related to Routes A and B.

According to the pKa of the hydrogen of the alcohol group compared with the α-hydrogen of the carbonyl group, **6** is expected to be thermodynamically more stable than **5**. DFT calculations confirmed this expectation, *i.e.*, **6a** is more stable by 9.7 kcal mol^-1^ than **5a**, and **6b** by 4.1 kcal mol^-1^ than **5b**. Therefore, **6** is the intermediate from the reaction of **2** and the base in both the fluoro and chloro compounds. Given that **5** has to be generated to form the target, we explored a likely TS structure of **TS_6-5_** connecting **6** and **5** (Appendix 1). The activation energies (Ea) were calculated to be more than 30 kcal mol^-1^ (39.4 kcal mol^-1^ for X=F and 33.0 kcal mol for X=Cl) as shown in Figure 1. Based on these calculations no target product will form under the proposed reaction conditions. Thus, our analysis concluded that Routes A and B are unlikely to generate the desired product.

Although the routes from the KOSP program adopted precursor **5** generated from **2**, it is also possible to produce the ion by use of the aldol condensation of 1-(2-halo-5-methylphenyl)ethanone and acetaldehyde using lithium diisopropylamide in THF, as also shown in Scheme 2, *via* an alternative method. This is a better route than Routes A and B to generate the alkoxide ion **5** because it avoids the synthesis of **2**. 

The C-O bond distances in **TS_5-1, _**where X=F or Cl, were 2.205 and 2.214 Å, respectively, and the activation energies were 9.1 and 12.1 kcal mol^-1^, respectively. However, **5** does not form in the reaction mixture because precursor **5** generates a complex **5’** with Li(THF)_2_^+^ as shown in Figure 1.

Two reactions are considered for **5’**: one is the S_N_Ar reaction to form the target, and the other is a proton transfer from the α-hydrogen atom of the carbonyl group to the alkoxyl oxygen atom to form **6’**. In both reactions, the Li(THF)^2+^ fragment reduces the nucleophilicity of the alkoxide oxygen. The alkoxide form **5’** was calculated to be more stable than **6’** with the anionic carbon (5.1 and 2.4 kcal mol^-1^ for X=F and Cl, respectively) because the coordination of the alkoxide oxygen to the Li(THF)^2+^ fragment releases a stabilization energy larger than that of the alcohol oxygen.

We succeeded in locating the TS for the reaction producing target **1’** as shown in Appendix 1. The C-O bond distances in **TS’_5-1_** for X=F and Cl were calculated to be 1.907 and 2.028 Å, respectively, and the activation energies to be 23.7 and 36.2 kcal mol^-1^, respectively. The corresponding barrier heights increase by 14.6 and 24.1 kcal mol^-1^ in comparison with those for **TS_6-5_**. The coordination of **5** to Li^+^ greatly reduces the nucleophilicity of the alkoxide oxygen atom. The H-C distance in **TS’_5-6_** was lengthened by 0.022 Å over that for **TS_6-5_** in both cases. **TS’_5a-1a_** is more stable by 12.5 kcal mol^-1^ than **TS’_5a-6a_**. Moreover, **1a’** is much more stable than **6a’**. These results suggest that the target is more likely to be synthesized using the alternative route. However, according to the calculated barrier heights the reaction will not take place at temperatures as low as -75 °C.

Water should be present in the THF solution of lithium diisopropylamide so that a water molecule may coordinate to Li^+^ in **5’** to form **5’’** (as shown in Appendix 1). The intermediate **5’’** was calculated to be slightly less stable (by 0.7 kcal mol^-1^) compared with **5’**. We found **TS”_5a-2a­_**_, _the TS structure in which the water molecule donates its proton to the alkoxide oxygen to form **2’**. In this structure, Li-OH_2_ and Li-O bond lengths were calculated to be 1.918 and 2.437 Å, respectively. Two O-H bond lengths were calculated to be 1.151 and 1.219 Å indicating a proton of the OH_2_ ligand is moving towards the alkoxide oxygen. The Ea of the process was calculated to be only 2.1 kcal mol^-1^ and the product **2’** was more stable by 0.7 kcal mol^-1^ than **5’**. It is considered that equilibration between **5’** and **2’** takes place in the reaction mixture and the S_N_Ar reaction of **5a’** does not proceed at a reaction temperature higher than 0 °C. Thus, the alternative route also fails to produce target **1**.

It was confirmed that the aldol condensation proceeded to form **5’** at -75 °C because the reaction yielded **2a**, as mentioned in the Experimental section. The calculations, including the counter ion effect, produced activation energies of more than 23.7 kcal mol^-1^. This barrier is high enough to prevent **5a’** from proceeding to generate the target at temperatures as low as -75 °C. Reactions conducted at 0 and 50 °C also produced **2a**, and other reaction mixtures generated no detectable target compound as anticipated from theoretical considerations.

### 2.2. Route C using the Mitsunobu Reaction

Schenk and his coworkers investigated the mechanism of the Mitsunobu reaction from a theoretical perspective [19]. This study used PH_3 _rather than PPh_3_, which eliminates any steric effect of the large substituents for the reaction. However, the steric effect is critical in order to avoid the side reactions of the α-hydrogen atoms in **3** of Route C. In the present study, we used the 6-31+G(d) level of theory because of the sizes of the molecules involved in the reaction. According to textbooks, the mechanism of the Mitsunobu reaction is very similar to Path B [20,21,22,23]. However, on the basis of functional groups in **3**, there are two other possible reactions.

**Scheme 3:**
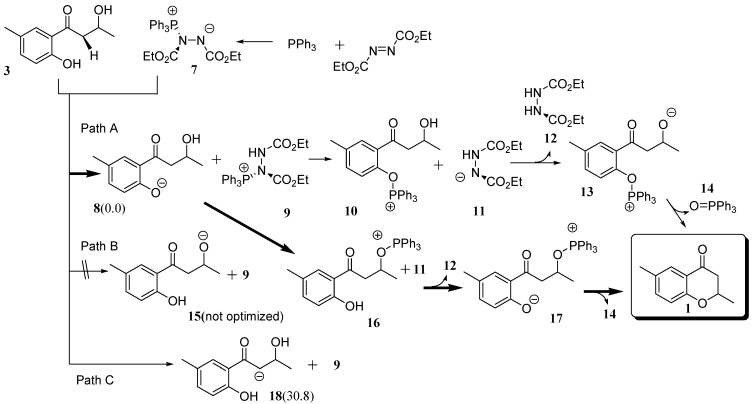
Plausible routes producing the target using Mitsunobu reaction.

**Figure 2:**
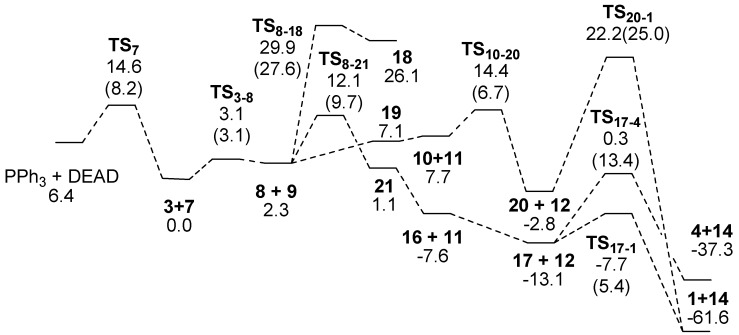
Energy correlation diagram (kcal mol^-1^) of the Mitsunobu reaction of **3**. Values in the diagram are energies relative to that of **3+7**. The numbersin parentheses are activation energies for each path.

In the Mitsunobu reaction, PPh_3_ first reacts with diethyl azodicarboxylate (DEAD) to form an intermediate **7**. Figure 2 displays an energy diagram related to this mechanism. Our calculations show that this reaction has a 8.2 kcal mol^-1^ barrier and is exothermic by 6.4 kcal mol^-1^. Almost all of the molecules in Route C consist of two fragments. The notation such as **8+9 **represents the optimized structure in which **8** interacts with **9**, in this case *via* hydrogen bonding. Hereafter, similar notations will be used for intermediates.

There are three reactive positions in **3**. The first is the hydroxyl group attached to the aromatic ring. Intermediate **7** extracts a proton to form **8** in Path A. The second is the hydroxyl group of the side chain, and the proton extraction from this group leads to the formation of the alkoxide ion **15** in Path B. It is necessary to consider the third possibility involving the α-hydrogen atom of the carbonyl group of the side chain. The loss of one α-proton leads to the formation of **18**. All extractions produce **9** as one of the products. In order to confirm which proton is extracted, geometry optimizations of the three anions were performed. Although we made an initial geometry of **15**, geometry optimization gave **8** as the most stable structure. Therefore, Path B cannot be practical. It was calculated that **8** is more stable by 30.8 kcal mol^-1^ than **18**. These results indicate that the reaction of **3** with **7** yields the intermediate **8+9**.

**Scheme 4:**
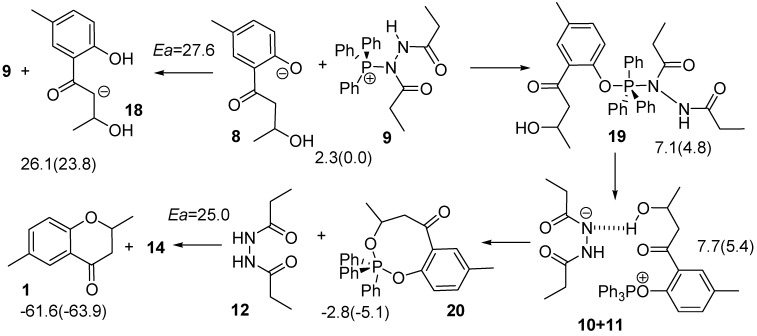
Reaction pathway from **8** to **1** *via* **19** and to **18**.

Three reactions were investigated to determine the likely outcome following the formation of **8** as shown in Eq. 2. The numbers in the equation indicate energies relative to **3+7** (**8+9**). TS structures obtained are shown in Appendix 2. The first is the reaction of the phenol anion extracting one of the α-hydrogen atoms to form **18** *via* **TS_8-18_** as shown in Scheme 4. It was calculated that this reaction has to overcome a barrier of 27.6 kcal mol^-1^ and **18** is less stable by 23.8 kcal mol^-1^ than **8+9**. The structure of **TS_8-18_** is shown in Appendix 2. This reaction is not considered any further because the other two reaction paths discussed below do not require overcoming such a high energy barrier.

The second reaction is between the phenol anion of **8** and the PPh_3_ fragment of **9** to form **19** with a hypervalent P atom and an O-P bond. This intermediate was calculated to be less stable by 4.8 kcal mol^-1^ than **8+9**. 

Before formation of **13** in Scheme 2, the P-N bond has to be cleaved to form **10** and **11**, and then fragment **11** successively changes its position adjacent to the hydroxyl group to form **10+11**. Fragment **11** extracts a proton from the hydroxyl group to form fragment **13**. However, it was confirmed that the reaction does not pass through **13**, but rather intermediate **20** with an eight membered ring. The H-N and H-O bond lengths in **TS_10-20_** in Appendix 2 were calculated to be 1.187 and 1.345 Å, respectively although the O-P bond length is rather long at 3.525 Å. We also found **TS_20-1_** formed **1+14**, the target **1** interacting with triphenylphosphine oxide (**14**). The calculated activation energy was rather high at 25.0 kcal mol^-1^. This is not a typical Mitsunobu reaction, and the reaction has an energy barrier higher than that for the third path. 

**Scheme 5:**
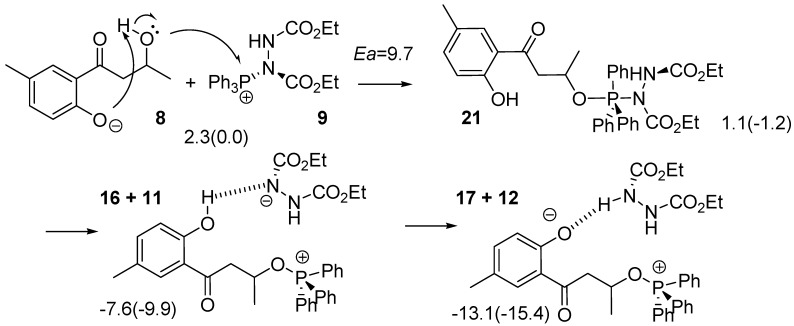
Reaction pathway from **8** to **17**.

The third reaction to be considered occurs *via* **16** as shown in Scheme 5. The numbers in the equation indicate energies relative to **3+7** (**8+9**). Because **15** is too unstable to be formed from **3**, **16** cannot be generated directly from **15**. However, an alternative route is feasible from **8+9**. Two changes in geometry occur simultaneously; the oxygen atom of the hydroxyl group of the side chain attacks the P atom of the PPh_3_ fragment of **9** and the anionic oxygen atom of **8** extracts the proton from the hydroxyl group. The O-H, H-O and O-P bond lengths were calculated to be 1.304, 1.123 and 2.448 Å, respectively. The IRC calculation using **TS_8-21_** indicated that the product of the reaction was not **16** but **21** with the P atom adopting a trigonal bipyramidal geometry. The barrier height for the TS was calculated to be only 9.7 kcal mol^-1^.

Due to a strong hydrogen bond between the hydroxyl group on the aromatic ring of **16** and the anionic nitrogen atom of **11**, **16+11** was calculated to be more stable by 8.7 kcal mol^-1^ than **21**. Therefore, the intermediate is considered to be easily decomposed to **16+11**. The succeeding reaction is an extraction of a phenol proton by **11** to form **17+12**. This reaction results in changing the hydrogen bond from O-H･･N to O･･H-N, and stabilizing the system by 5.5 kcal mol^-1^. As the barrier height for **TS_8-21_** is lower by 2.3 kcal mol^-1^ than that of **TS_10-20_**, the reaction *via* **TS_8-21_** mainly proceeds to form **17+12**. The intermediate **17** is a typical intermediate of a Mitsunobu reaction. 

**Scheme 6:**
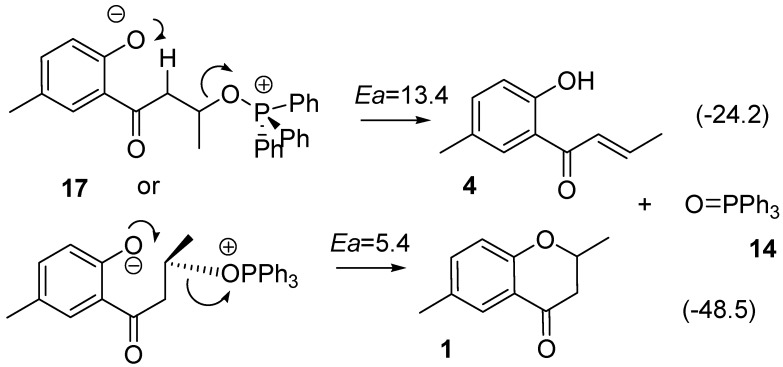
Reaction pathway forming the target **1** and the subproduct **4** from **17**.

The anionic oxygen of **17** attacks the carbon atom adjacent to the OPPh_3_ moiety in **17** and this reaction leads to the formation of **1** *via* **TS_17-1_** as shown in Scheme 6. Values in parentheses are energies relative to **17**. In the TS, the C--O and C--O=PPh_3_ bond lengths were calculated to be 2.298 and 1.834 Å, respectively, and the central carbon is almost flat, as shown in Appendix 2. Although the anionic oxygen attacks the tertiary carbon, **TS_17-1_** has typical geometrical features of TSs for S_N_2 reactions. This reaction accompanies inversion at the carbon atom. Such a change in geometry is usually seen in the Mitsunobu reaction. The activation barrier was calculated to be only 5.4 kcal mol^-1^ and a very large heat of reaction (such as 48.5 kcal mL^-1^). 

It is noteworthy that there is another TS, **TS_17-4_**, in which the phenol oxygen anion extracts an α-hydrogen atom with concomitant loss of the O=PPh_3_ fragment to form **4**. The O-H, H-C and C-O bond lengths in the TS were calculated to be 1.460, 1.219 and 1.917 Å, respectively. The structure has the typical features of TSs in geometry for the E2 mechanism. The **TS_17-4_** is less stable by 8.0 kcal mol^-1^ than the **TS_17-1_**. 

**Scheme 7:**
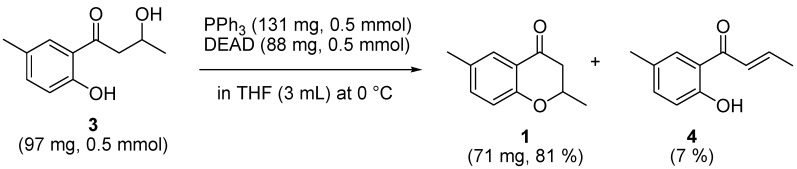
Yields of the main and the sub products using Mitsunobu reaction.

Hodgetts *et al.* had already examined this synthetic route and succeeded in producing the target **1** in good yield (81%). Enone **4** was observed to be a subproduct of the synthetic experiments, although the energy difference between the two TSs is larger than that expected from the experiments (7%). Baldwin and coworkers have previously reported this subproduct [24].

### 2.3. Route D Using the Michael Reaction

**Scheme 8:**
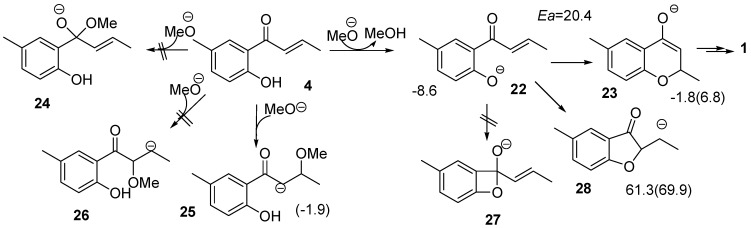
Plausible reactions considered for the Michael reaction.

The addition of a nucleophile to activated double bonds, as seen in Route D, is referenced in the Michael reaction. A methoxide ion in dry methanol extracts a phenol proton of **4** to form **22**, followed by the addition of this anion to the double bond to form **23** in Scheme 8. Numbers in the equation indicate energies relative to **4 **(**22**). The keto-enol equilibrium leads to the formation of **1**. To our knowledge, this is a novel synthetic route to produce the target. 

**Figure 3:**
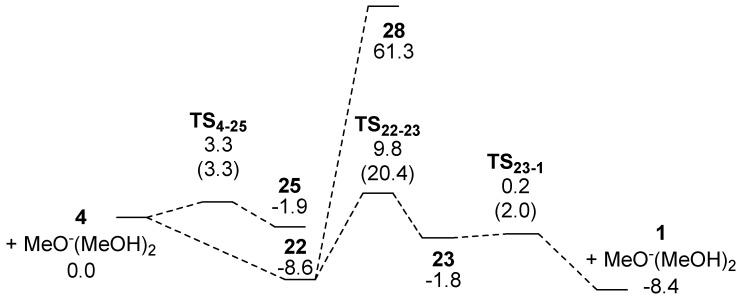
Energy correlation diagram (kcal mol^-1^) and structures related to the Michael reaction for the target **1**.

Although only the phenol fragment of **4** has a proton that the base extracts, two more functional groups exist for the reaction with the methoxide ion. One is the carbonyl group, in which the anion attacks its carbon to form the tetrahedral intermediate **24**. The other is the double bond on the side chain, and its reaction with the anion produces **25** or **26**. Theoretical calculations optimized only one stable structure **25**. In these calculations, two methanol molecules interacting with the lone pair orbitals of the methoxide oxygen were included, as shown in Appendix 3. As **25** was calculated to be less stable by 10.5 kcal mol^-1^ than **22** at the B3LYP/6-31+G(d) level of theory as shown in Figure 3, extracting a proton from the phenol fragment occurs more readily than addition of the methoxide ion to the carbonyl carbon. 

**Scheme 9:**
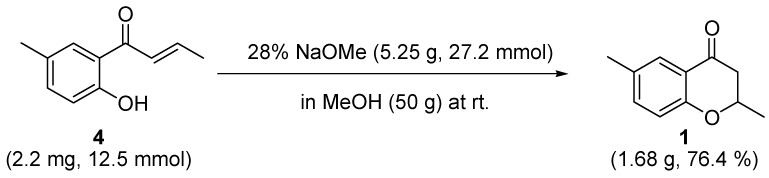
Yields of the target using the Michael reaction.

There are three feasible reactions for **22** to create a ring. The first one generates **27** with a four membered ring by a reaction of the carbonyl carbon with the anionic oxygen charge. However, the small ring is expected to cause this product to be very unstable. Thus, no stable structure was obtained. Secondly, the double bond reacts with the anion fragment to produce **28** with a five membered ring. It was calculated that **28** is less stable by 69.9 kcal mol^-1^ than **22**. By contrast, the third reaction generates **23** with a six membered ring, which is the precursor for **1**, with a barrier of 20.4 kcal mol^-1^ and is endothermic by 6.8 kcal mol^-1^. A C-O bond is attempting to form in the **TS_22-23_** as shown in Appendix 2 but the distance between the atoms was calculated to be 1.948 Å. Enolate **23** has to extract a proton from MeOH, and accept it at the carbon adjacent to the carbonyl group to form the target. The low activation energy, as little as 2.0 kcal mol^-1^, suggests this process proceeds smoothly. Product **1** is more stable by 6.6 kcal mol^-1^ than **23**. Therefore, the DFT calculations revealed that the Michael reaction using **4** is a promising strategy for synthesizing target **1**. A synthesis experiment was then performed in dry MeOH, which gave **1** in an overall yield of 76.4%, albeit as a mixture of enantiomers (Scheme 9).

## 3. Experimental

### 3.1. Density Functional Theory (DFT) Calculations

Geometry optimizations including TSs were carried out using Density Functional Theory (DFT) calculations from the Gaussian03 program [25] at the B3LYP/6-31+G(d) level of theory for Routes A, B and D, or the B3LYP/6-31G(d) level of theory [26,27,28] for Route C. The GaussView03 [29] program was used for molecular modeling. The intrinsic reaction coordinates (IRCs) [30] were calculated to analyze all of the mechanisms in detail. The vibration frequencies were calculated to confirm that the obtained geometries would be stable for the TS structures.

### 3.2. Synthesis of 1-(2-fluoro-5-methylphenyl)-3-hydroxy-1-butanone *(**2a**)*

A solution of 2’-fluoro-5’-methylacetophenone (152.2 mg, 1.0 mmol) in THF (0.5 mL) was added to a solution of diisopropylamine (111.3 mg, 1.1 mmol) and butyllithium (1.60 M in hexane, 0.69 mL, 1.1 mmol) in THF (1 mL), and then stirred for 1 h at –78 °C. The mixture was added to a solution of acetaldehyde (84.0 µL, 1.5 mmol) in THF (0.5 ml), stirred for 2 h at –78 °C, quenched with saturated ammonium chloride (5 mL), and then extracted with dichloromethane (10 mL × 5). The combined extracts were dried over anhydrous magnesium sulfate and concentrated under reduced pressure. The residue was chromatographed on a silica gel with hexane – ethyl acetate (1/1 v/v) as an eluant to give 1-(2-fluoro-5-methylphenyl)-3-hydroxy-1-butanone (155.6 mg, 79%) as a colorless oil. 

### 3.3. Synthesis of 1-(2-hydroxy-5-methylphenyl)but-2-en-1-one *(**4**)*

Anhydrous aluminium chloride (3.92 g, 29.4 mmol) was added in small portions to a solution of *p*-methylanisole (3.66 g, 30 mmol) and crotonoyl chloride (3.06 g, 29.3 mmol) in carbon disulfide (146 mL) at 20~30 °C. After stirring for 2 h, additional anhydrous aluminium chloride (3.92 g, 29.4 mmol) was added all at once, and the mixture was stirred at 35~40 °C until the evolution of hydrogen chloride had ceased (about 3 h). The reaction mixture was then poured slowly into a mixture of concentrated hydrochloric acid (10 mL) and ice water (150 g). The organic layer was separated, washed with water and saturated sodium chloride, dried, and then concentrated under reduced pressure. The residue was chromatographed on a silica gel with hexane–ethyl acetate (10/1 v/v) as an eluant to give 1-(2-hydroxy-5-methylphenyl) but-2-en-1-one (4.4 g, 83%) as a yellow oil. The spectral data for this compound matched those previously reported.

### 3.4. 2,6-dimethylchroman-4-one *(**1**)*

A solution of 1-(2-hydroxy-5-methylphenyl)but-2-en-1-one (2.20 g, 12.5 mmol) and 5% aqueous sodium hydroxide (147 mL) was stirred at room temperature for 16 h. The precipitate was filtered, washed with water, and dried to give a pale brown solid (2.18 g) in which the unreacted material was detected by TLC. The precipitate was dissolved in a mixture of 28% sodium methoxide (5.25 g, 27.2 mg) and dry methanol (50 g), and stirred for 5 h at room temperature. The mixture was acidified to pH 5 with 1 N hydrochloric acid and concentrated under reduced pressure. The residue was chromatographed on silica gel with hexane–ethyl acetate (10/1 v/v) as an eluant to give 2,6-dimethylchroman-4-one (1.68 g, 76%) as a yellow solid. The spectral data for this compound matched those previously reported.

## 4. Conclusions

In the present study, we have analyzed the reaction mechanisms for four synthetic routes for 2,6-dimethylchroman-4-one using the B3LYP/6-31+G(d) or B3LYP/6-31G(d) level of theory. Four reactions were selected from among the 21 synthetic routes created by the program KOSP. The DFT calculations suggested that two routes using the S_N_Ar mechanism would not generate the desired product. It was also confirmed from theoretical calculations that an alternate route using the aldol condensation could not produce the target compound. These findings are consistent with our experimental data which have done after the present calculations.

The theoretical analysis clearly showed that the target should be obtained from Route C. This route has already been used to synthesize the target with a good yield of 81%. However, the mechanism of this Mitsunobu reaction has not been clarified from a theoretical standpoint. The DFT calculations indicated the possibility of generating a byproduct, which was observed in Hodgetts experiments. Our proposed mechanism explains the formation of both the main product as well as the observed byproduct.

Theoretical examinations confirmed that Route D using the Michael reaction is also a feasible route for obtaining the desired target. Our synthesis experiments produced the target with an overall yield of 76.4%, although no attempt was made to fully optimize the reaction conditions.

**Scheme 10:**
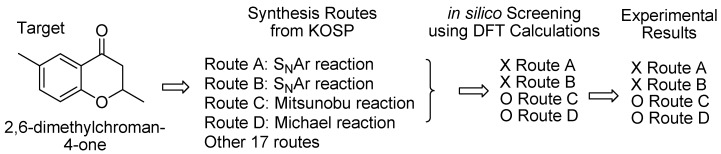
Results of *in silico* synthesis development of the target.

Scheme 10 displays the results of “*in silico* synthesis development” of the target including the comparison between the results of *in silico* screenings and synthesis experiments. We performed a theoretical analysis of the reaction mechanisms for Routes A, B and D, and concluded that only the last route was worth attempting experimentally. It is important to point out that the KOSP program created these synthetic routes investigated here, and no experiments had been conducted prior to our theoretical investigations. The KOSP program also created Route C, which Hodgetts *et al*. used to synthesize **1** prior to our calculations. However, the mechanism of this reaction had not been investigated.

Synthetic organic chemists can create synthetic routes for targets, but cannot give answers to their feasibility without performing the actual experiments. Theoretical analyses of the reaction mechanisms for these synthetic routes are considered to be a valuable tool for examining the potential means of obtaining the desired product. We can use theoretical calculations to narrow down the number of experiments that have to be conducted (e.g., from potential routes generated using the KOSP program). Thus, the combination of computational chemistry and cheminformatics offers a new way of helping to develop novel synthetic routes for target compounds. Nonetheless, real experiments still have to be performed and optimized in order to create new synthetic schemes for industrial applications.

## Figures and Tables

**Appendix 1:**
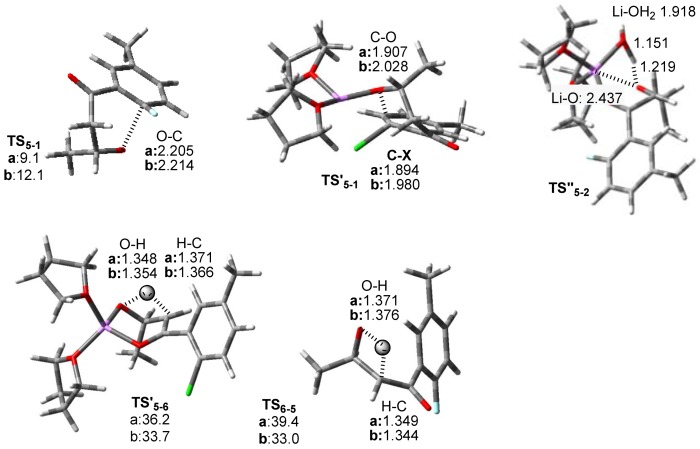
3D structures related to Figure 1. Lengths in the Figure are in Å units.

**Appendix 2:**
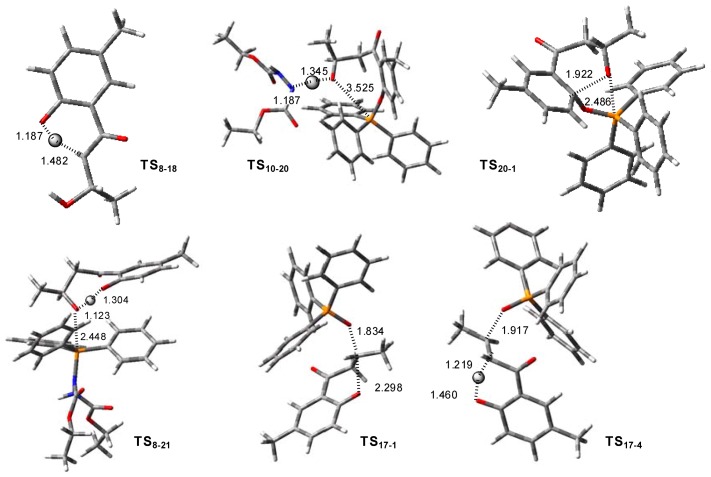
3D structures related to Figure 2. Lengths in the Figure are in Å units.

**Appendix 3:**
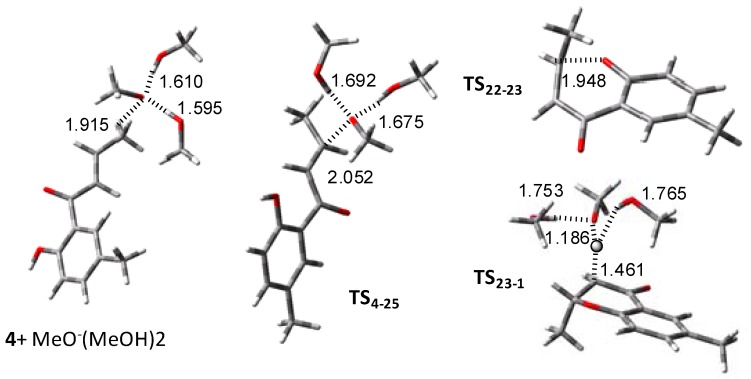
3D structures related to Figure 3. Lengths in the Figure are in Å units.
